# Population Estimation and Demographic Characteristics of Free-Roaming Dogs on Kasetsart University Kamphaeng Saen Campus, Thailand: Implications for Rabies Control

**DOI:** 10.3390/biology14070808

**Published:** 2025-07-03

**Authors:** Tita Phiriyaphokhai, Thitiwan Patanasatienkul, Tipsarp Kittisiam, Suwicha Kasemsuwan, Kansuda Leelahapongsathon

**Affiliations:** 1 Saraburi Provincial Livestock Office, Department of Livestock Development (DLD), Mueang Saraburi 18000, Saraburi, Thailand; tita0575m@gmail.com; 2 Department of Veterinary Public Health, Faculty of Veterinary Medicine, Kasetsart University, Kamphaeng Saen 73140, Nakhon Pathom, Thailand; suwicha.k@ku.ac.th; 3 Centre for Veterinary Epidemiological Research, Atlantic Veterinary College, University of Prince Edward Island, Charlottetown, PE C1A 4P3, Canada; thitiwan.patanasatienkul@gmail.com; 4 Department of Health Management, Atlantic Veterinary College, University of Prince Edward Island, Charlottetown, PE C1A 4P3, Canada; 5 Department of Population Medicine, Ontario Veterinary College, University of Guelph, Guelph, ON N1G 2W1, Canada; tipsarp.k@gmail.com

**Keywords:** free-roaming dogs, population estimation, dog population management, negative binomial regression, Thailand

## Abstract

Free-roaming dogs (FRDs) pose significant public health and animal welfare challenges globally, particularly in developing nations, where they serve as major reservoirs for zoonotic diseases, especially rabies. Determining the population size of FRDs, their demographic characteristics, and the factors influencing population size is essential for effective dog population management, which constitutes a critical component of rabies control programs. This study aimed to estimate the size and describe the characteristics of the FRD population and evaluate temporal and spatial variations in FRD sightings on the Kasetsart University Kamphaeng Saen Campus, Thailand. Factors influencing population size were assessed using a negative binomial regression model. The campus area was divided into 52 accessible blocks for dog-counting purposes. In 2018 and 2019, each block was surveyed once annually for a comprehensive census. In 2021, each block was surveyed on six occasions, three in the morning and three in the evening, using a photographic recapture method. Dogs sighted were photographed, and their characteristics and spatial coordinates were recorded. The total population sizes of FRDs on campus were documented as 529, 577, and 531 in 2018, 2019, and 2021, respectively. Intact female dogs predominantly constituted the majority of the FRD population. Regression analysis indicated a significant association between the number of FRDs within a block and the number of intact adult female dogs. The percentage of dogs sighted per occasion was significantly higher during evening surveys compared to morning surveys. This study proposes a practical census approach that combines total block counts with photographic recapture to estimate the population of FRDs. The findings offer valuable insights for developing effective dog population management strategies and planning rabies vaccination campaigns.

## Simple Summary

Free-roaming dogs are commonly seen in many communities, including at Kasetsart University Kamphaeng Saen Campus, Thailand. A major concern is that these dogs can spread zoonotic diseases, especially rabies, which is fatal to both animals and humans. A better understanding of the population size and characteristics of free-roaming dogs, as well as the factors influencing their numbers, is crucial for planning effective population control and rabies prevention programs. In this study, the campus was divided into 52 accessible blocks. A census of free-roaming dogs on the campus was conducted by directly counting individual dogs in all blocks. To estimate the actual population, multiple surveys were carried out in the same block, and all dogs were photographed to avoid double-counting. Factors influencing population size were also examined. The results showed that more dogs were observed in the evening than in the morning. Most dogs were unsterilized females, and their presence was associated with the total number of dogs in each area. This study provides a practical approach for estimating free-roaming dog populations and highlights the importance of sterilizing female dogs as part of effective rabies control efforts.

## 1. Introduction

Free-roaming dogs (FRDs) are domestic dogs that are not confined or restrained. They constitute approximately 75% of the global dog population [1]. FRDs represent owned dogs, strays, community dogs, and feral dogs with minimal human interaction [2,3]. The high concentration of FRDs poses numerous global public health and animal welfare challenges, particularly in developing countries [2,4,5,6]. FRDs contribute to incidents of dog bites [7,8], road accidents [5,9], and environmental issues such as noise pollution, fecal contamination, and scavenging through garbage [5,10]. Due to limited access to food and veterinary care, FRDs often suffer from poor health [4,5]. According to the World Health Organization (WHO) report, these dogs play crucial roles in transmitting various zoonotic diseases, notably rabies [11,12,13].

Rabies is one of the most life-threatening issues globally, with an estimated 60,000 deaths annually, over 40% of which are children in Asia and Africa [14]. This issue is also prevalent in Thailand, where rabies remains endemic. Almost 99% of human rabies cases are attributed to dog bites [14], and FRDs contribute to the persistence of the disease in many countries. In Thailand, most dogs roam freely, regardless of ownership status. In urban areas, owned dogs are generally confined, but in rural regions, they are often allowed to roam freely and reproduce uncontrollably [15]. Many stray dogs are abandoned in public spaces, such as temples and universities, where they may become community dogs, cared for by individuals who provide food and water [15]. However, these dogs often lack access to veterinary care, including vaccination, particularly against rabies and reproductive control. The abundance of FRDs is largely due to a lack of owner education, irresponsible pet ownership, and inadequate reproductive control measures [16].

To control FRD populations, the capture-and-kill policies have been prohibited by the WHO, prominent researchers, and animal protection organizations due to being inhumane and ineffective [5]. Instead, reproductive control, a core element of dog population management (DPM), is acknowledged as a humane and effective method for limiting FRD population growth and turnover [17]. Currently, both permanent methods, such as surgical and chemical sterilization, and temporary options, like contraceptives, are available for reproductive control [18,19], with surgical sterilization being the most commonly used approach [19]. The concept of DPM is now recognized as a vital strategy for addressing issues related to FRDs by controlling their population size and turnover rate, improving their health and welfare, and increasing the accountability of dog owners or caretakers [5,17,18]. Moreover, the World Organisation for Animal Health (WOAH) recommends DPM as a crucial component of rabies control programs [20].

In order to design sustainable and effective DPM programs and rabies control initiatives, it is necessary to have accurate knowledge of the size of the dog population [21,22,23,24]. This includes documenting dog characteristics such as sex, age, and reproductive status (neutered/spayed or intact). These data are essential for allocating resources, including budgets, human resources, and equipment, and determining appropriate intervention strategies, such as surgical sterilization or rabies vaccination campaigns. Additionally, understanding the factors influencing the population size is crucial for planning interventions effectively.

FRD overpopulation presents a significant challenge in various communities, including Kasetsart University (KU) Kamphaeng Saen (KPS) Campus in Nakhon Pathom Province, Thailand. Complaints from staff and students regarding incidents such as dog bites, road accidents, noise pollution, and scavenging led to the initial efforts to capture aggressive dogs and house them in a shelter. These dogs were neutered/spayed and vaccinated against rabies. However, the shelter quickly reached its capacity with fewer than a hundred dogs, leaving many others roaming freely and contributing to population growth. To address the issue, several capture–neuter–return (CNR) programs and vaccination campaigns were implemented. However, the absence of baseline data on population size and characteristics limited the effectiveness of these efforts. Meanwhile, the growing FRD population continued to cause inconveniences and raise public health concerns among staff and students. Therefore, the objectives of this study were to estimate the size and describe the characteristics of the FRD population, identify factors associated with population size, and evaluate temporal and spatial variations in FRD sightings on the Kasetsart University Kamphaeng Saen Campus.

## 2. Materials and Methods

### 2.1. Study Site

The study was conducted on KU KPS Campus in Nakhon Pathom Province, Thailand, which includes approximately 12.72 km^2^ of buildings and agricultural fields. The campus is frequented by more than 15,000 people per day, and it features three main accessible gates, with fences and canals serving as its borders.

**Figure 1:**
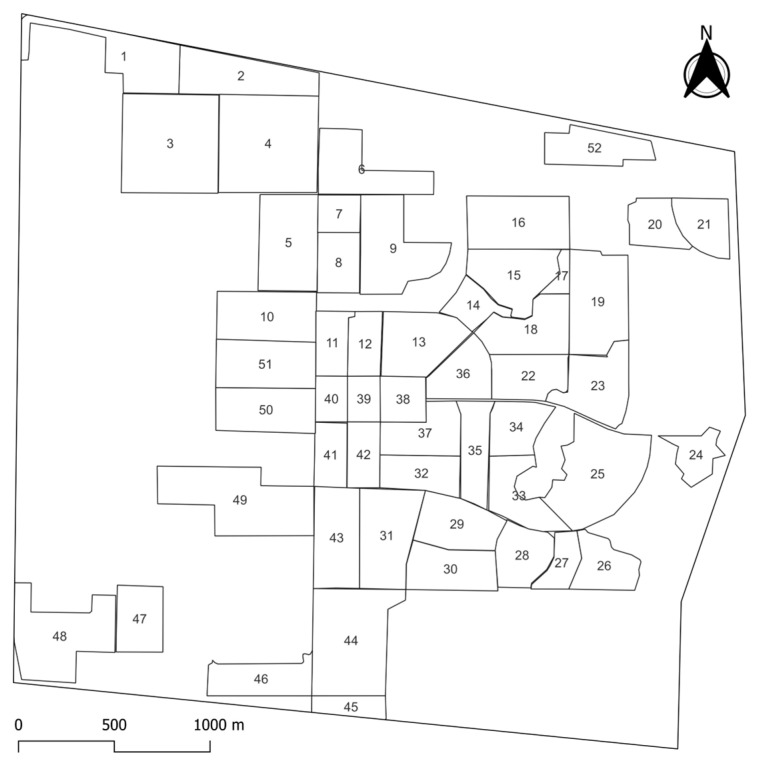
Map of Kasetsart University, Kamphaeng Saen campus, displaying all 52 accessible blocks.

The criteria from World Animal Protection (formerly World Society for the Protection of Animals; WSPA) [1] were adopted to divide the campus area into blocks. Moreover, more criteria were included as follows: (1) each block must be accessible by car, motorbike, or on foot; (2) each block must have a minimum size of 0.025 km^2^; (3) there are no restrictions on entering each block; and (4) roads and natural boundaries (e.g., lakes or canals) must serve as borders for each block. The campus area was divided into 52 accessible blocks (Figure 1) using Google My Maps [25]. Each block consisted of a part or an entire area of faculties, offices, agricultural fields, and residential zones. Notably, access was possible to 52 blocks, covering approximately 5.56 km^2^ (approximately 40% of the total campus area). Due to restrictions and the presence of dense agricultural fields (e.g., sugar cane fields), pond areas, and overgrown regions, observation and investigation of FRDs were not possible in other areas.

### 2.2. Study Population

In this study, FRDs are defined as dogs observed in public areas that were not confined or under direct human control. This included owned dogs that were allowed to roam freely, and stray dogs. All FRDs sighted in 52 blocks were counted. Dogs in the other areas were not included in this study due to the aforementioned limitations. It was assumed that the FRD population on the campus was closed.

### 2.3. Data Collection

Data on the FRD population surveyed in 2018 and 2019 on KU KPS Campus from a previous study [26] were used in this study. In that previous study, the total block count method was employed to conduct a census of the FRD population. Observations were carried out in January 2018 and between January to February 2019. Following this approach, two teams, each consisting of two individuals, were assembled and employed both car and on-foot surveys to conduct comprehensive investigations twice daily (6.30–7.30 a.m. and 4.30–5.30 p.m.). Each of the 52 blocks was surveyed once annually, and each survey occasion involved approximately 1 h of observation per block. Individual records were collected for dog characteristics, including sex, age, physical characteristics, GPS coordinates, and photographs.

In August 2021, a census of the FRD population on the campus was conducted using the total block count method with photographic recapture. This recapture technique requires surveying the same area using consistent procedures on two or more occasions to determine the actual population count within that area. Specifically, this method involves photographing all individual animals in the study area and using these photos to identify the same animals across different survey periods. Observations were conducted twice daily in the morning (7:00–9:00 a.m.) and evening (4:00–6:00 p.m.) to maximize the likelihood of detecting the highest number of dogs in the area. The 52 blocks were grouped into four sections of 13 contiguous blocks each. A team of two to four researchers systematically surveyed each section of 13 blocks over three consecutive days. The first section was surveyed on days 1 to 3, the second on days 4 to 6, the third section on days 7 to 9, and the final section on days 10 to 12. Consequently, each block was examined on six separate occasions (twice daily for three consecutive days). Surveys were conducted using cars, motorcycles, and foot patrols, with each block requiring approximately 15 min to explore.

During each observational survey, researchers recorded the visual characteristics of all identified dogs, whether previously documented or newly observed. The observed dogs were photographed using a digital camera (Nikon COOLPIX P1000, Nikon (Thailand) Co., Ltd., Phra Nakhon Si Ayutthaya, Thailand) and mobile phones, capturing at least two angles (front and side) for identification. Data were recorded using the Epicollect5 mobile application [27], as summarized in Table 1. Each dog record documented various physical attributes, including coat color, natural markings or scars, tail shape, and other distinctive features, along with age, sex, and reproductive status. Additionally, the spatial coordinates (GPS coordinates) of each dog were collected. The classification of free-roaming dogs on the campus comprised two categories: pups and adults. Dogs were categorized as pups if they were observed relying on their mother for sustenance for approximately the first four months of life [1]. Sex determination was conducted by observing the genitalia of the dogs whenever feasible. In cases where it was difficult or impossible to ascertain their sex, they were categorized as having an “unknown” sex. Regarding reproductive status, male dogs were classified as intact if their testes were present, while the absence of testes indicated spayed or neutered status. Female dogs were presumed to be intact unless a spaying mark was visible on the thigh or information regarding their status was provided by caretakers. Throughout the study, researchers maintained a distance from the dogs and refrained from interfering with their activities.

### 2.4. Data Analyses

#### 2.4.1. Estimations of FRD Population Size

**Figure 2:**
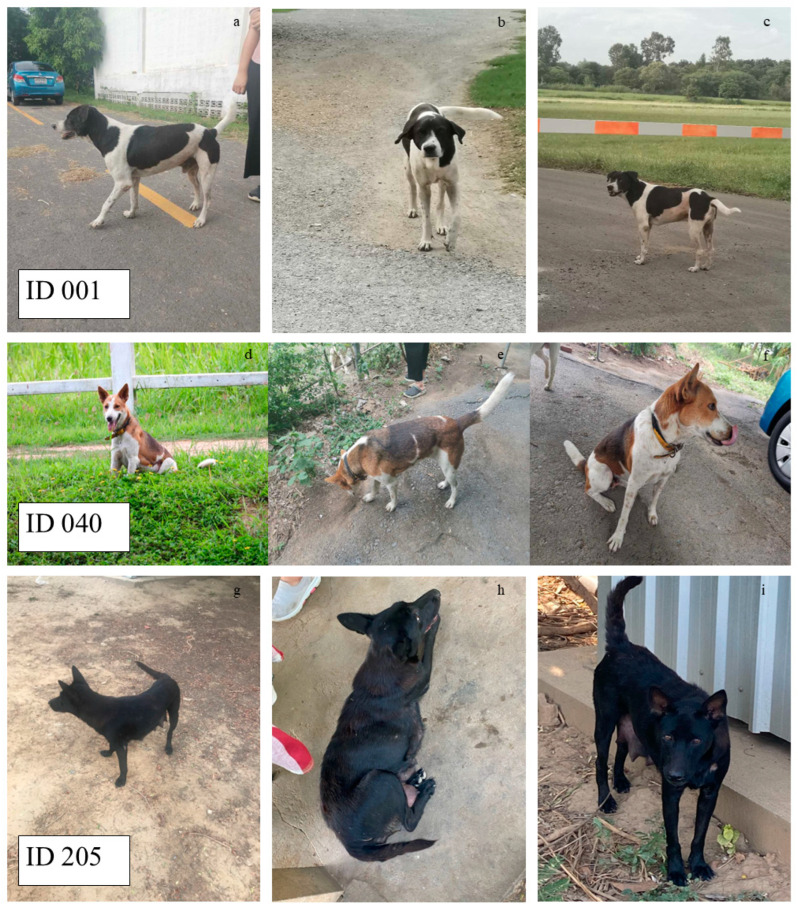
Examples of dog identification during the survey period in 2021: ID 001 (**a**–**c**) located in block 48 (male, neutered, adult, two-colored: black and white, cow-liked pattern coat, drop ears, and saber tail). ID 040 (**d**–**f**) located in block 49 (female, spayed, adult, multicolored, yellow and black collared, erect ears, and saber tail), and ID 205 (**g**–**i**) located in block 1 (female, intact, adult, monocolored: black, enlarged mammary glands, erect ears, and saber tail).

The FRD population sizes in 2018, 2019, and 2021 were estimated by directly counting all the dogs observed across the 52 blocks. Observational data from 2021 were exported from Epicollect5 for the purpose of individual dog identification. Subsequently, all records relating to free-roaming dogs in 2018, 2019, and 2021 were cleaned and summarized in Microsoft Excel [28]. Every dog sighted on each occasion was appropriately labeled. Identification of each dog was accomplished by referencing the photographs with respect to the date and block. It was noted that some dogs were observed in more than one block during the survey period in 2021. The block where dogs were most frequently sighted during the six occasions was selected and documented in the spreadsheet. Individual dogs were identified based on their coat colors, distinctive features, and tail shapes for each respective year (Figure 2). Additionally, special attention was paid to dog characteristics and physical appearances to avoid duplicate counts. Only unique individual dogs were included in the annual population count. The frequencies and percentages of FRDs and their characteristics, including age, sex and reproductive status, were presented for each year.

#### 2.4.2. Multivariable Analyses of Factors Influencing Population Size

Multivariable analyses were carried out using Stata (version 17.0) [29]. The negative binomial regression model and the negative binomial mixed-effects regression model were used to assess the factors influencing the FRD counts in each block. The dataset exhibits a hierarchical structure with two levels. Each of the 52 blocks was surveyed annually over three years, resulting in 156 observations at level 1. The number of FRDs sighted annually within each block was treated as level 2, nested within the blocks. Six variables were evaluated as predictors of the frequency of FRDs (Table 2). These predictors included the following categorical variables: (1) presence of food source, recorded as either present or absent based on the existence of a canteen or restaurant within each block; (2) year of observations, categorized as 2018, 2019, or 2021. The continuous variables were as follows: (1) block area size (km^2^); (2) number of intact adult female dogs per block per year; (3) number of intact adult male dogs per block per year; (4) proportion of building area within each block. Initially, all predictors were individually evaluated in univariable negative binomial models for their association with the number of FRDs, using a liberal cut-off for inclusion (*p*-value < 0.2). Subsequently, significant predictors identified from this initial screening were incorporated into a multivariable negative binomial model. A backward stepwise approach was then manually applied to remove predictors with the highest *p*-value first. Predictors with *p*-value < 0.05 were retained in the final model. The same set of predictors was also used in the negative binomial mixed model to assess variability among blocks.

#### 2.4.3. Analysis of FRD Sightings Variation

Data from six survey occasions (three in the morning and three in the evening) in 2021 were used to evaluate variation in FRD sightings. The number of occasions on which individual dogs were sighted was summarized based on all six survey occasions, only the three morning occasions, and only the three evening occasions. The percentage of dogs sighted per occasion in each block was calculated by dividing the number of dogs sighted in each occasion by the total number of dogs in that block. These percentages were then compared across survey periods (morning, evening) and block characteristics, including building area level (categorized based on the proportion of building area within each block: low < 0.078; high ≥ 0.078), and presence of food source (no, yes). The comparison of means for the percentage of dogs sighted per occasion, across survey periods and block characteristics, was conducted using a generalized least-squares (GLS) method in the “nlme” package [30], followed by Tukey’s post hoc tests in R version 4.4.3 [31].

## 3. Results

### 3.1. FRD Population Sizes and Demographic Characteristics

**Figure 3:**
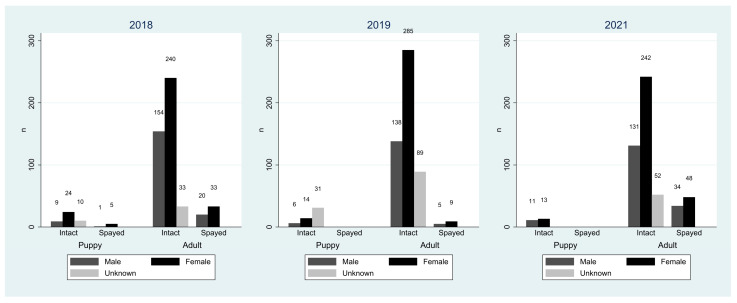
The histogram illustrates the number of sighted dogs categorized by age, reproductive status, and sex, respectively, from 2018 to 2021.

In 2018, 2019, and 2021, the KU KPS Campus recorded 529, 577, and 531 FRDs, respectively. Each year, the number of intact male dogs observed was 163, 144, and 142, while the number of neutered male dogs was 21, 5, and 34, respectively. Female dogs categorized as intact were 264, 299, and 255, while those categorized as spayed were 38, 9, and 48, respectively. Over the three-year observation period, intact female dogs averaged 49.9% of all dogs observed (range: 48.0–51.8%). Intact male dogs averaged 27.5% (range: 25.0–30.8%). Unknown-sex dogs averaged 12.9% (range: 8.1–20.8%). A summary of the demographic characteristics is presented in Table 3 and Figure 3. The majority of FRDs on the campus were adult intact females across all three years. Data on free-roaming dogs on the KU KPS Campus in 2018, 2019, and 2021, including identification, sex, reproductive status, age, and location by block and GPS coordinates, are presented in Supplementary Table S1.

**Figure 4:**
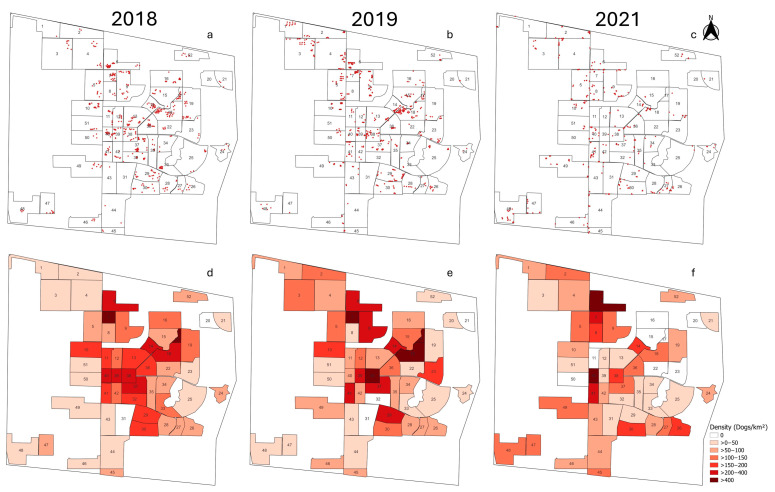
Spot maps and choropleth maps showing the locations and density of FRDs within the 52 accessible blocks of Kasetsart University, Kamphaeng Saen Campus. Panels (**a**–**c**) show the locations of FRDs in 2018, 2019, and 2021, respectively. Panels (**d**–**f**) show the density of FRDs in 2018, 2019, and 2021, respectively.

Spot maps and choropleth maps were generated to visualize the spatial distribution and density of FRDs in 2018, 2019, and 2021 across the 52 accessible blocks of Kasetsart University, Kamphaeng Saen Campus (Figure 4). In 2018, blocks 7 and 17 exhibited the highest densities of FRDs, with 403.60 and 661.88 dogs/km^2^, respectively. Block 7 contained both built-up areas and agricultural fields, while block 17 was a student residential area. In 2019, in addition to blocks 7 and 17, high densities of FRDs were also found in blocks 18 and 38, with 426.02, 458.22, 423.58, and 493.22 dogs/km^2^, respectively. Block 18 was another student residential area, whereas block 38 served as a central campus space where people frequently gathered for socializing and physical exercise in the evenings. In 2021, blocks 6 and 40 showed the highest densities of FRDs, with 1045.43 and 400.99 dogs/km^2^, respectively. Block 6 included sugarcane fields and some built-up areas. During our observation, more than 30 dogs were sighted in a single location within block 6, contributing to the high density shown in Figure 4f. Numerous feeding sites with leftover dog food, provided by caretakers, were also observed along the road adjacent to block 6. Block 40 comprised faculty buildings and a restaurant. The location of each dog in the 2021 survey was assigned based on the block where it was most frequently sighted during the six survey occasions. However, our observations revealed that some dogs moved between blocks, though their movement was generally limited to adjacent or neighboring blocks.

### 3.2. Factors Associated with Population Size

Following univariable model assessments for the number of FRDs using a liberal inclusion cut-off (*p*-value < 0.2), block area size, number of intact adult female dogs, and intact adult male dogs were included in the multivariable analysis (Table 4). A likelihood ratio test, comparing the mixed-effect negative binomial regression to the standard negative binomial regression, yielded χ^2^ < 0.01 and *p* < 0.01, indicating no significant block-level variability. Consequently, the standard negative binomial regression was used as the final model for this dataset. Table 5 presents the results of both the standard negative binomial regression and negative binomial mixed regression models.

The likelihood ratio test for α = 0 yielded χ^2^ = 141.33 and *p* < 0.001, indicating a significant difference from zero. The degree of overdispersion, calculated using 1 + αμ = 1 + (0.17 × 10.49) = 2.78, provided evidence of overdispersion. Goodness-of-fit tests, including the Pearson’s test (χ^2^ = 139.26, df = 154, *p* = 0.80) and deviance test (χ^2^ = 180.68, df = 154, *p* = 0.07) suggested that the model fit the data. Therefore, the number of intact adult female dogs was significantly associated with increased FRD counts within the block.

### 3.3. FRD Sightings Variation

The numbers of FRDs sighted across different numbers of sighting occasions based on all six survey occasions, the three morning occasions, and the three evening occasions, are presented in Table 6. Of the 531 FRDs recorded in 2021, the majority were sighted only once (n = 173, 32.58%). A total of 135 dogs (25.42%) were not observed during morning surveys, while 92 dogs (17.33%) were absent from evening surveys.

Differences in the mean percentage of dogs sighted per occasion in each block across survey periods, building area level, and presence of food source are presented in Table 7. A significant difference was found between morning and evening survey periods (*p* < 0.01). Tukey’s post hoc test indicated that the percentage of dogs sighted in the evening was significantly higher than in the morning (*p* < 0.01).

## 4. Discussion

In this study, we estimated the FRD population size at Kasetsart University Kamphaeng Saen Campus through three censuses conducted in 2018, 2019 and 2021, and described demographic characteristics. We also employed the negative binomial regression model to investigate factors associated with the number of FRDs. These findings provide valuable baseline information to support the planning of dog population management programs and rabies vaccination campaigns on campus. Furthermore, this study offers a practical framework for estimating FRD populations in other regions using total block counts combined with photographic recapture.

World Animal Protection recommends the block count method, which involves dividing the study area into blocks to estimate FRD population size [1]. This method is particularly suitable for visual observations in areas with sparse vegetation [32] and is widely used in wildlife studies, having been applied to various species, including alpine chamois [33], blue sheep [34], serows [32], sika deer [35,36], and red and roe deer [37]. The block count method has been employed in previous studies in southeastern Iran [38] and Dhaka, Bangladesh [21], where sample blocks were randomly selected in accordance with World Animal Protection guidelines to estimate FRD total populations within those areas. In this study, we adapted World Animal Protection guidelines by using all blocks to enumerate the total number of dogs present on the campus. The block count method offers several advantages, including enabling comprehensive surveys and assisting in the identification of dogs associated with specific locations. Additionally, the use of adjacent blocks allows surveyors to conduct efficient investigations within the study’s time constraints, which limited each survey session to two hours. However, it is essential to note that variations in block size and the movement of dogs between blocks present potential challenges. To mitigate potential issues of recounting the same dogs, we implemented dog identification techniques, including photographic records, physical appearance records, and point location data.

The study was conducted within a university campus characterized by low-density built-up areas and agricultural fields, which was divided into 52 accessible blocks for survey purposes. Some FRDs moved between their assigned and adjacent blocks, likely influenced by their home range and the availability of food sources. Studies from Chile and Australia reported average FRD home ranges of 0.65 km^2^ and 0.40 km^2^, respectively [39,40], with maximum distances traveled up to 1.05 km [39], whereas the largest block in our study was only 0.26 km^2^. In addition to canteens and restaurants, food was also provided by caretakers at feeding sites scattered throughout the campus. Our observations indicated that FRDs often moved to adjacent blocks to feed and returned afterward. However, when food was available within their assigned blocks, they tended to remain in those areas. Areas with at least one caretaker were more likely to consistently provide food resources, attracting greater numbers of FRDs to those locations [41]. High FRD densities have been associated with areas of high human population density [42] and consistently available food sources [43]. Furthermore, studies in rabies-endemic countries have shown that FRDs tend to reside near buildings and roads, regardless of urban or rural setting [44]. Proximity to buildings offers food, water, and shelter provided by humans, while roads facilitate movement and accessibility to food sources [45,46].

Numerous studies have estimated the size of the FRD population using mark-resight or mark-recapture methods [21,47,48,49], along with photographic sight–resight or photographic recapture techniques [38,43,50,51,52,53,54,55,56,57,58]. These approaches are considered more cost-effective, faster, and easier to manage, while also minimizing potential risks during handling [38]. Revisiting the same area multiple times enhances the likelihood of obtaining the actual population size. Previous studies have typically conducted surveys either once a day [38,55,56] or twice daily, during mornings (between 5:00 and 9:00 a.m.) and evenings (between 4:00 and 7:00 p.m.) [43,50,51,53,54,58] to coincide with periods of increased FRD activity [57,59] and optimal lighting conditions for photography [52,58]. Some studies have revisited areas multiple times, ranging from three to five times [50,51,53,54,55,56,58]. In this present study, each block was visited six times in 2021, three times in the morning and three times in the evening, to improve the accuracy of population estimates. A significantly higher percentage of dogs was sighted in the evening compared to the morning, which may be attributed to caretakers feeding them during that time, leading to increased activity and visibility compared to the morning. FRDs typically engage in behaviors such as foraging and territory marking [59]. A study conducted in Indigenous communities in northern Australia found that dogs roamed the furthest in the evening, coinciding with human social activities as people returned from work or school and engaged in neighborhood interactions [54]. For future studies involving FRD studies, it is important to consider the activities of relevant communities when planning surveys to enhance observational accuracy.

The total number of FRDs on the campus experienced slight fluctuations over the years, ranging from 529 to 577 dogs. Similarly, a study on the University of São Paulo campus found stability in the dog population size, potentially due to factors such as a high mortality rate (from accidents or disease), dog rescues and re-homing, and the capture and relocation of dogs to university animal shelters [43]. One or a combination of these factors may have influenced the population size in this study. According to the university dog shelter team, some dogs were adopted by faculty and students, and approximately 20 to 50 dogs were captured annually and relocated to the university shelter on the campus and other locations. However, the exact number of captured dogs was not officially documented. No evidence of deceased dogs or carcasses was found during the study period. In contrast, a study conducted in the municipality of Divinópolis, Minas Gerais, Brazil, where FRDs were not removed from the area, observed an increase in the FRD population size over time due to dog abandonment by owners [60]. Other studies have reported substantial FRD growth rates, including 9.0% in Kenya [61], 6.5% in Zimbabwe [62], 9.0% in Chile [63], and 8.0% in Tanzania [64]. Therefore, given these trends, the population size of FRDs in this study area is expected to increase over time in the absence of intervention.

According to the results, there were more female dogs than male dogs, which differs from other studies [55,60,61,64,65,66]. Previous research has shown that male dogs were more likely to display aggressive behavior than females [67], and that intact males were more aggressive than castrated males [67,68]. Consequently, we hypothesized that the higher number of females was due to the campus shelter’s capture team targeting aggressive male dogs for removal to shelters. Additionally, there was a noticeable difference in the percentages of puppies observed between the 2018–2019 and 2021 surveys. In 2018 and 2019, the percentages of puppies were 9.3% and 8.8%, respectively, whereas in 2021, the proportion declined to 4.5%. A previous study estimating the FRD population in India indicated percentages of puppies ranging from 6.5% in rural areas to 7.7% in urban areas [53]. Additionally, research conducted near the Great Indian Bustard Wildlife Sanctuary reported puppy percentages ranging from 4.5% to 18.2% [58], which aligns with the findings of our study. Environmental factors have been shown to influence FRDs’ reproduction [69,70,71,72,73,74,75]. In the tropical climate of India, the peak mating period occurs in late monsoon (October), with the peak births observed in winter (December and January) [74,75]. Given Thailand’s similar tropical climate, it is plausible that FRDs in this region may exhibit comparable reproductive patterns. For future population surveys, it is recommended that studies be conducted during the winter months to accurately assess the peak proportion of puppies.

The model identified the number of intact adult female dogs as the sole significant predictor associated with an increase in population size. A study conducted in Tanzania noted that free-roaming female dogs could begin reproducing as early as six months of age [64], with previous research showing they could give birth annually [9,76]. The average litter size ranged from 4.6 to 6.0 puppies [63,64,70,74,77]. Thus, focusing sterilization on female dogs could be an effective strategy for controlling or reducing the FRD population on the campus. Several studies have demonstrated that the effective reduction in FRD populations is primarily attributable to the sterilization of female dogs [38,78,79]. The International Companion Animal Management Coalition (ICAM) recommended achieving a 70% annual sterilization rate for female dogs to ensure an effective DPM program [17]. Moreover, reducing the population size can facilitate achieving 70% vaccination coverage against rabies, enhancing accessibility and cost-effectiveness of disease prevention efforts. This approach also helps decrease turnover rates by mitigating high death rates due to infections from giving birth. As a result, it aids in maintaining vaccination coverage even if the population size remains constant [66,80]. Historically, DPM programs in Thailand have prioritized sterilization of male dogs due to time constraints and cost-effectiveness. Future research should further explore the effect size of the targeted sterilization of female dogs to determine whether the benefits of population control outweigh the costs compared to the current male-leaning approach.

This study had several limitations. Firstly, during the 2021 survey, campus activity was reduced due to COVID-19 control measures. Most classes were conducted online and students were largely absent from campus. However, faculty and staff remained present, resulting in a level of activity comparable to summer and winter breaks. Secondly, we were unable to assess the population dynamics due to the absence of data on death rates and the number of dogs captured and relocated to the shelter. For future studies, we recommend coordinating with the campus shelter’s capture team to systematically record the number of dogs captured and their characteristics. Thirdly, the negative binomial regression model used in this study was primarily based on statistical criteria rather than relative biological significance. Future research should explore human-related variables such as socioeconomic status, cultural factors, and social beliefs of the local community to better understand their influence on the FRD population.

## 5. Conclusions

This study demonstrated the application of total block counts combined with photographic recapture to estimate the population size of FRDs on the KU KPS Campus in Thailand. The findings indicated that the FRD population size exhibited slight variations over time. Importantly, the number of intact female dogs was identified as a significant contributor to the population size within each block. In addition, FRDs were more frequently sighted in the evening than in the morning surveys. Therefore, DPM programs should prioritize the sterilization of female dogs, particularly in areas with high FRD densities to enhance program effectiveness.

## Figures and Tables

**Table 1:** The data collection of each FRD that was recorded using Epicollect5.

Data	Categories	Method of Estimation
GPS coordinates	Latitude and longitude	GPS recording in Epicollect5 app.
Block	The block number	Google My Maps current location
Sex	Male/Female/Unknown	Observation of reproductive organs
Reproductive status	Intact/Spayed or Neutered	Male: Observation of reproductive organs Female: Observation of circular brands on thighs and/or caretaker interviews
Age	Puppy/Adult	Observation of dogs if they depend on their mothers
Physical characteristics and other prominent appearances	Coat color: Multicolored/Two-colored/ Mono-colored Prominent appearances: Natural marks and/or scars Ear shape: Erect/Drop/Semi-erect Tail shape: Bobbed/Docked/Curly/Whip/ Saber	Observation

**Table 2:** Descriptive statistics of variables used.

Variables	n (%)	Mean	Variance	SD	Median	IQR
Number of FRDs in each block	156	10.49	164.03	12.81	7	4–14.5
Presence of food source	156					
No	132 (84.62)					
Yes	24 (15.38)					
Year of observations 2018 2019 2021	156 52 (33.33) 52 (33.33) 52 (33.33)					
Block area size (km^2^)	156	0.11	0.003	0.06	0.09	0.06–0.13
Number of intact adult female dogs	156	5.24	25.90	5.09	4	2–7
Number of intact adult male dogs	156	2.88	17.22	4.15	2	1–4
Proportion of building area within each block	156	0.09	0.007	0.08	0.078	0.03–0.17

**Table 3:** Total number of sighted free-roaming dogs and characteristics of free-roaming dogs within KU KPS Campus, Thailand in 2018, 2019, and 2021.

Number of Dogs: n (%)	Year		
	2018	2019	2021
Total dogs	529	577	531
Age			
Adults	480 (90.7%)	526 (91.2%)	507 (95.5%)
Puppies	49 (9.3%)	51 (8.8%)	24 (4.5%)
Sex			
Male	184 (34.8%)	149 (25.8%)	176 (33.1%)
Female	302 (57.1%)	308 (53.4%)	303 (57.1%)
Unknown	43 (8.1%)	120 (20.8%)	52 (9.8%)
Reproductive status			
Intact	470 (88.8%)	563 (97.6%)	449 (84.6%)
Neutered or spayed	59 (11.2%)	14 (2.4%)	82 (15.4%)

**Table 4:** Predictors with *p*-value less than a liberal cut-off for inclusion (*p* < 0.2) that were included in multivariable analysis.

Variables	Coefficient (SE)	*p*-Value
Block area size (km^2^)	35.56 (18.26)	0.053
Number of intact adult female dogs	2.27 (0.07)	<0.001
Number of intact adult male dogs	2.83 (0.10)	<0.001

**Table 5:** The results of negative binomial regression compared to negative binomial mixed regression using the number of dogs in each block as an outcome.

Variables	Negative Binomial Regression ^a^		Negative Binomial Mixed Regression ^b^	
	Coefficient (SE)	*p*-Value	Coefficient (SE)	*p*-Value
Intercept	1.25 (0.08)	<0.001	1.25 (0.08)	<0.001
Number of intact adult female dogs	0.15 (0.01)	<0.001	0.15 (0.01)	<0.001
Block variance σ	-	-	7.52 × 10^−31^ (3.72 × 10^−16^)	-

^a^ Akaike Information Criterion (AIC) = 881.90; ^b^ AIC = 881.90.

**Table 6:** The number of FRDs sighted in 2021 across different numbers of sighting occasions based on all six survey occasions, the three morning occasions, and the three evening occasions.

Number of Sighting Occasions	Number of Dogs Sighted: n (%)		
	Six Occasions	Three Morning Occasions	Three Evening Occasions
0	-	135 (25.42%)	92 (17.33%)
1	173 (32.58%)	168 (31.64%)	184 (34.65%)
2	86 (16.2%)	104 (19.59%)	131 (24.67%)
3	67 (12.62%)	124 (23.35%)	124 (23.35%)
4	67 (12.62%)	-	-
5	76 (14.31%)	-	-
6	62 (11.68%)	-	-

**Table 7:** Mean ± SEM (Standard Error of Mean) of percentage of dogs sighted per occasion in each block across survey periods, building area level, and presence of food source.

Variables	Mean ± SEM	95% CI	*p*-Value
Survey period			<0.01
Morning	51.7 ± 4.0	43.8–59.7	
Evening	64.4 ± 4.0	56.4–72.3	
Building area level			0.74
Low	56.6 ± 5.0	46.5–66.7	
High	59.5 ± 4.1	51.2–67.8	
Presence of food source			0.53
No	56.8 ± 2.7	51.3–62.2	
Yes	59.3 ± 5.9	47.4–71.2	

## Data Availability

The data supporting the findings of this study are available from the authors upon request and were used with permission from Kasetsart University, Kamphaeng Saen campus.

## Supplementary Materials

The following supporting information can be downloaded at: https://www.mdpi.com/article/10.3390/biology14070808/s1, Table S1: Free-roaming dogs on Kasetsart University Kamphaeng Saen Campus, Nakhon Pathom, Thailand, in 2018, 2019 and 2021, showing identification (DogID), sex, reproductive status (reprostatus), age, and location by block and GPS coordinates.

## Abbreviations

The following abbreviations are used in this manuscript:

FRD	Free-roaming dog
WHO	World Health Organization
DPM	Dog population management
WOAH	World Organisation for Animal Health
KU	Kasetsart University
KPS	Kamphaeng Saen
CNR	Capture–neuter–return
GLS	Generalized least-squares
WSPA	World Society for the Protection of Animals
ICAM	International Companion Animal Management Coalition
